# Markets and Morals: An Experimental Survey Study

**DOI:** 10.1371/journal.pone.0127069

**Published:** 2015-06-01

**Authors:** Julio J. Elias, Nicola Lacetera, Mario Macis

**Affiliations:** 1 Universidad del CEMA, Av. Córdoba 374, C1054AAP, Buenos Aires, Argentina; 2 University of Toronto, Institute for Management and Innovation, 3359 Mississauga Road North, Mississauga, ON, Canada, L5L 1C6; 3 Johns Hopkins University, 100 International Drive, Baltimore, Maryland, 21202, United States of America; University of Reading, UNITED KINGDOM

## Abstract

Most societies prohibit some market transactions based on moral concerns, even when the exchanges would benefit the parties involved and would not create negative externalities. A prominent example is given by payments for human organs for transplantation, banned virtually everywhere despite long waiting lists and many deaths of patients who cannot find a donor. Recent research, however, has shown that individuals significantly increase their stated support for a regulated market for human organs when provided with information about the organ shortage and the potential beneficial effects a price mechanism. In this study we focused on payments for human organs and on another “repugnant” transaction, indoor prostitution, to address two questions: (A) Does providing general information on the welfare properties of prices and markets modify attitudes toward repugnant trades? (B) Does additional knowledge on the benefits of a price mechanism in a specific context affect attitudes toward price-based transactions in another context? By answering these questions, we can assess whether eliciting a market-oriented approach may lead to a relaxation of moral opposition to markets, and whether there is a cross-effect of information, in particular for morally controversial activities that, although different, share a reference to the “commercialization” of the human body. Relying on an online survey experiment with 5,324 U.S. residents, we found no effect of general information about market efficiency, consistent with morally controversial markets being accepted only when they are seen as a solution to a specific problem. We also found some cross-effects of information about a transaction on the acceptance of the other; however, the responses were mediated by the gender and (to a lesser extent) religiosity of the respondent—in particular, women exposed to information about legalizing prostitution reduced their stated support for regulated organ payments. We relate these findings to prior research and discuss implications for public policy.

## Introduction

Most modern societies rely on markets for organizing exchanges. It is widely recognized that the price mechanism achieves greater efficiency compared to other methods of allocating resources, by conveying information on the relative scarcity of goods and services, thereby guiding buyers and sellers to make optimal economic decisions [1,2,3,4]. In spite of this, the application of a market or price mechanism to certain transactions is sometimes opposed on moral grounds (and legally banned) even in the absence of negative externalities, and even when the parties involved in a trade might arguably benefit from it [5]. For example, in the United States payments to organ donors are illegal, and so are markets for sex (with the exception of certain parts of Nevada). The opposition to these transactions rests in part on a desire to protect vulnerable individuals, e.g. from exploitation or coercion [6]. However, the aversion often comes from the idea that these trades would compromise sacred and inviolable values, corrupt morals, or generate a sense of distaste [7,8,9].

Prohibiting some of these transactions has costs. Life insurance contracts, for instance, were once illegal because they were seen as gambles against God; they now create value for millions of people, and are viewed as a form of “institutionalized altruism” [10]. Similarly, the idea of an all-volunteer paid army was long rejected in the United States, despite arguments showing its efficiency [11]. The prohibition of payments to people who give their organs contributes to the growing gap between organ demand and supply, with severe effects on lives lost and financial costs [12]; recent studies estimate that payments between $15,000 and $30,000 would close the gap between demand and supply [13]. Banning some trades may also lead to the formation of illegal markets, which, in turn, entail further costs such as violence [14]; for instance, recent evidence shows that legalizing indoor prostitution reduced sexually transmitted diseases and violence against sex workers [15].

Moral constraints contribute to defining a society’s culture, thus they have a value on their own that might justify the economic costs that these constraints imply. These values, moreover, change slowly [16]. However, differences over time and across societies in what trades are considered repugnant raise the question of how repugnance forms and evolves. In particular, technological progress and the increased availability of data and methods to measure the efficiency of certain transactions might lead to changes in attitudes about their acceptability if their costs and benefits are revealed. But do attitudes about which trades are acceptable depend at least in part on calculations about their material costs and benefits as opposed to reflecting deeply-held, “sacred” values [17]?

In previous research we developed an experimental survey instrument through which we found that providing information on the estimated positive effects of regulated payments to organ donors increased significantly the support among U.S. residents for the legalization of these payments. We concluded that well-supported information on costs and benefits of a specific transaction can affect whether individuals believe that the trade should be allowed [18]. The finding were specific to the case of payments for organs; auxiliary tests where other subjects were expressing their support for legalized indoor prostitution or slavery contracts, after having read information about these activities and the potential effect of their legalization, showed no aggregate response to information.

The findings from that study motivated a number of additional questions to fully understand the mechanisms through which scientific information affects attitudes about the acceptance of certain market transactions. A first question is whether providing *general* information on the welfare properties of prices and markets modify attitudes toward repugnant trades. It has been argued that stimulating a market-oriented approach in people may lead to a relaxation or even corruption of their moral values [9,19]. However, empirical evidence either in support or against this claim is scant, and especially so from socially relevant contexts.

A second question is whether having additional knowledge on the benefits of using a price mechanism in a *specific* context affects attitudes toward market-based transactions in *another* context, i.e. whether there is a cross-effect of information. The emergence of evidence on the beneficial role of a price mechanism in a morally controversial setting might also change beliefs about the acceptability of such a mechanism in another context. The cross-effect, if any, could also go in the opposite direction; it is possible that thinking about markets in one context increases the “repugnance” for another set of transactions.

We chose to focus on two of the activities considered in our previous work: payments for organs and indoor prostitution. The baseline approval rates for these two activities showed a similar degree of moral controversy, and the legalization of both trades is currently being debated [20,21]. Moreover, the legalization of these two transactions, albeit in different ways, concerns the definition of legal markets for the human body (or parts of it); thus, at least in theory, spillover effects of information may be expected. Finally, our prior study showed that, whereas the responses to information in the case of organ payments were very similar across subgroups of the sample population, stark heterogeneities emerged, in the case of legalized indoor prostitution, between subjects of different genders and religiosity. In particular, women and subjects who stated to be religious (as opposed to atheist/agnostic), who in previous studies displayed lower support rates for legalized prostitution [22,23,24,25], *reduced* their approval for the legalization of indoor prostitution in the U.S. after reading a summary of the evidence of some benefits from its legalization [18]. For certain individuals, therefore, being exposed to information on the costs and benefits of legalizing morally controversial trades might trigger even a deeper opposition for that particular trade or even for others. Recent research found a similar effect, with information about vaccine safety reducing intent to vaccinate among respondents who initially displayed high levels of concern about the vaccine’s side effects [26].

In the next section we describe our research methodology; we then present our results and discuss their implications.

## Materials and Methods

The project was approved by the Research Ethics Board of the University of Toronto (protocol 30238) and by the Institutional Review Board of Johns Hopkins University (protocol 1991). The consent by the subjects was written and provided at the beginning of the experimental procedure. The boards listed above approved this procedure. We recruited 5,324 subjects over three waves in September, November, and December 2014 via Amazon Mechanical Turk (mTurk), an Amazon Web Service platform that allows reaching a large number of individuals to perform tasks online and is increasingly used for surveys and experiments [27,28]. The subjects agreed to participate in the study (described as a “computerized questionnaire”) and to receive $0.75 upon its completion; the tasks altogether took about 5 minutes, making the implied hourly wage slightly above the mTurk average for North America. Participants were then randomly assigned to one of the following conditions:

- *Control*: For subjects in this experimental condition, we first elicited their attitudes toward legalizing payments for either organ donors or their families in case of deceased donation, or for legalized indoor prostitution; then, we administered a survey with questions about their demographics and socio-economic characteristics. Details on the survey questions and structure can be found in the Supporting Information.

In all of the treatment conditions described below, participants were first informed that they would be shown a text, followed by a reading comprehension question. The various texts (of about 500 words each) are reported in S1 Text.

- *Organs text and approval for legalized indoor prostitution*: Individuals in this treatment condition were provided a text that reported information about the current organ supply shortage in the United States, and described a number of proposals that have been advanced or, in some cases, implemented to reduce such shortage, with references to the academic studies advancing or evaluating these proposals. These included kidney exchange programs [29] as well as studies estimating the effects of introducing monetary compensation for donors [13]. The remainder of the survey was the same as that the one presented to the control group, with elicitation of attitudes toward legalizing indoor prostitution in the U.S. and a socio-economic survey.
- *Prostitution text and approval for payments to organ donors*: This experimental condition was symmetrical to the previous one: the text provided included information about recent academic work that showed that the legalization of indoor prostitution in a U.S. state led to large reductions in sexual violence and certain sexually transmitted diseases [15]. Subjects were then surveyed about the approval for payments for organ donors or their families.
- *Market text*: In this condition, subjects received a text with general (not context-specific) arguments concerning the beneficial welfare properties of market exchanges, as commonly described in standard economic textbooks. We then split this sample to gather attitudes toward legalizing organ payments and support for the legalization of indoor prostitution.

To preserve anonymity and allay concerns for social desirability bias in the responses, the questions about support for organ payments or legalized indoor prostitution were asked with the “Item Count Technique” (ICT). The ICT is based on not asking a question directly (e.g., “Would you support the implementation of regulated payments for organ donors or their families?”); instead, respondents are shown a set of statements and are asked to indicate how many apply to them. The control group is given N “neutral” statements (i.e., non-sensitive in nature and not related to the research topic) whereas the treatment group receives N+1 sentences, of which N are the same as for the control group, and the additional item is the one of interest. Thus the researcher cannot infer whether a given respondent answered positively or negatively to a specific item; only the total number of items that apply to an individual is identifiable. This preserves the privacy of the respondents and, together with the anonymity of the online survey, reduces the concern that the subjects provide what they perceive to be the “socially correct” answer. In our case, the hypothetical framework might lead to a downward bias in the estimates of treatment effects if most respondents believed that paying for organs or allowing a market for sex were generally considered morally wrong and felt that that was the “right” answer. The random assignment of subjects to the experimental conditions, the choice of statements that are not perfectly correlated (to avoid individuals agreeing with all or none of them, thus effectively revealing their opinion on each statement), and the use of a large enough sample size, make the difference in the average counts between subjects receiving N+1 and N statements a valid estimate of the share of individuals in the population under study to which the statement of interest applied [30,31].

**Fig 1:**
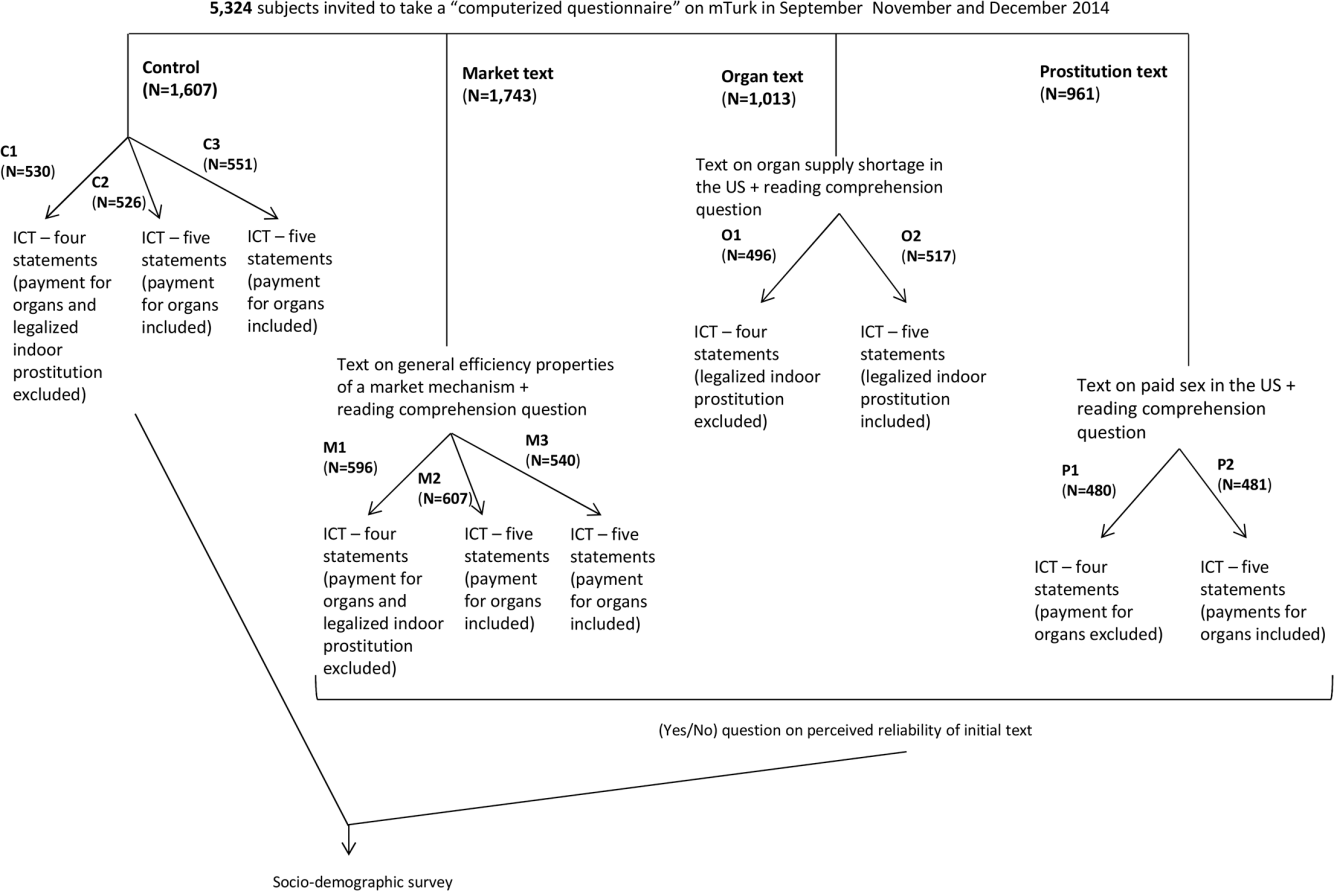
Experimental design.

Within each of the experimental conditions, subjects were thus further randomly divided in two subgroups of roughly equal size: one receiving four statements, and one receiving five statements. The fifth statement was a sentence indicating that the respondent would support the establishment of a regulated system of payments for organs (or legalized indoor prostitution) and, overall, the other statements were chosen such that the majority of respondents would be unlikely to agree with either all of them or none of them (see the Supporting Information for details). The distribution of responses (Table 1) confirms that we achieved this goal. The sample sizes for each treatment condition (at the level of the split between being assigned four and five statements) were chosen so that we would be able to detect, with 5% confidence and 80% power, differences of at least 10 percentage points in support rates. Smaller differences, even if precisely estimated, would be of limited interest. The experimental design is represented in Fig 1. Table 2 reports statistics on the socio-demographics, showing balance across the experimental conditions. We had no “attrition” in the survey, with the subjects who started it also getting to its completion. This is important because it allows us to rule out the possibility of selective attrition (i.e., attrition driven by the treatment), which would make the interpretation of the results problematic. In addition, we found no statistically significant differences in the distribution of socio-demographic characteristics across the different treatment groups (Chi-squared tests never reject the null hypothesis of equal distributions, and only a handful of pairwise t- tests indicated statistically significant differences across experimental conditions). The Supporting Information also includes additional details on the characteristics of the subjects, and a comparison with the socio-demographic characteristics of the US population (our sample is younger, and it over-represents Caucasians, college-educated individuals, and individuals who declare liberal political views compared to the US population) (S1 Table and S2 Table).

**Table 1:** Distribution of number of statements with which subjects reported to agree.

Number of statements that apply to subject	4 statements received (N = 2,102)	5 statements received (N = 3,222)	Total (N = 5,324)
0	3.8%	2.6%	3.1%
1	22.8%	11.2%	15.8%
2	50.1%	33.1%	39.8%
3	21.7%	36.4%	30.6%
4	1.6%	14.5%	9.4%
5		2.2%	1.4%

**Table 2:** Descriptive statistics on individual characteristics collected from the survey, overall and by experimental condition.

	Women	Caucasian	Afr. Amer.	Other ethn.	Married	College	Christian	Conservative	Liberal	Obs.
No text, 4 statements	46.8%	74.7%	9.1%	16.2%	31.1%	49.6%	45.1%	17.0%	47.5%	530
No text, 5 statements (Organs)	50.6%	77.0%	6.5%	16.5%	35.4%	47.7%	50.2%	19.8%	46.6%	526
No text, 5 statements (Prostitution)	52.3%	77.7%	8.0%	14.3%	36.8%	49.7%	47.4%	21.5%	41.1%	551
Market text, 4 statements	50.5%	77.5%	9.2%	13.3%	36.2%	51.8%	45.8%	22.1%	42.6%	496
Market text, 5 statements (Organs)	51.9%	77.9%	9.1%	13.0%	34.1%	47.3%	45.6%	19.8%	45.0%	517
Market text, 5 statements (Prostitution)	51.3%	79.1%	6.9%	14.1%	33.0%	50.7%	45.4%	19.4%	49.7%	480
Organs text, 4 statements	52.8%	77.2%	7.7%	15.1%	31.9%	50.4%	45.6%	17.2%	49.3%	481
Organs text, 5 statements (Prostitution)	49.3%	80.3%	6.0%	13.7%	33.7%	48.7%	42.6%	17.1%	49.4%	596
Prostitution text, 4 statements	52.1%	77.7%	7.1%	15.2%	35.6%	54.6%	46.3%	20.6%	46.0%	607
Prostitution text, 5 statements Organs	52.8%	79.0%	8.3%	12.7%	35.6%	48.0%	51.8%	19.3%	46.8%	540

The econometric models used to estimate support rates for legalized indoor prostitution or organ payments take the following form:

$$Y_{i}=\beta_{C4}+\beta_{C5}D_{iC5}+\beta_{T4}D_{iT4}+\beta_{T5}D_{iT5}+\gamma X_{i}+\varepsilon_{i},$$ (1)

where *Y*_*i*_ is the count of statements with which subject *i* is in agreement, and the binary indicators *D*_*iC*5_, *D*_*iT*4_ and *D*_*iT*5_ take a value of 1 if subject *i* is assigned to the control group with five statements, to a treatment group with four statements, or to a treatment group with five statements, respectively (we estimate the model separately for T = “market text”, T = “organs text”, and T = “prostitution text”). Thus, the estimate ${\hat{\beta }}_{C4}$ indicates the average number of agreed statements for the control-group subjects who received four statements. The estimate ${\hat{\beta }}_{C5}$ reports the difference between the number of agreed statements by subjects in the control group who had five statements and those who received four statements, or the estimated share of subjects who would support the establishment of regulated payments for organs or legalized indoor prostitution. Similarly, the difference ${\hat{\beta }}_{T5}-{\hat{\beta }}_{T4}$ is the estimate of the approval rate for payments to organ donors or legalized indoor prostitution for those in one of the treatment groups. Finally, $({\hat{\beta }}_{T5}-{\hat{\beta }}_{T4})-{\hat{\beta }}_{C5}$ estimates the difference in approval rate between the treatment group and the control group, our main treatment effect of interest. The vector *X*_*i*_ includes covariates derived from the socio-economic survey responses, as well as an indicator for the wave in which the experiment was conducted.

## Results

**Fig 2:**
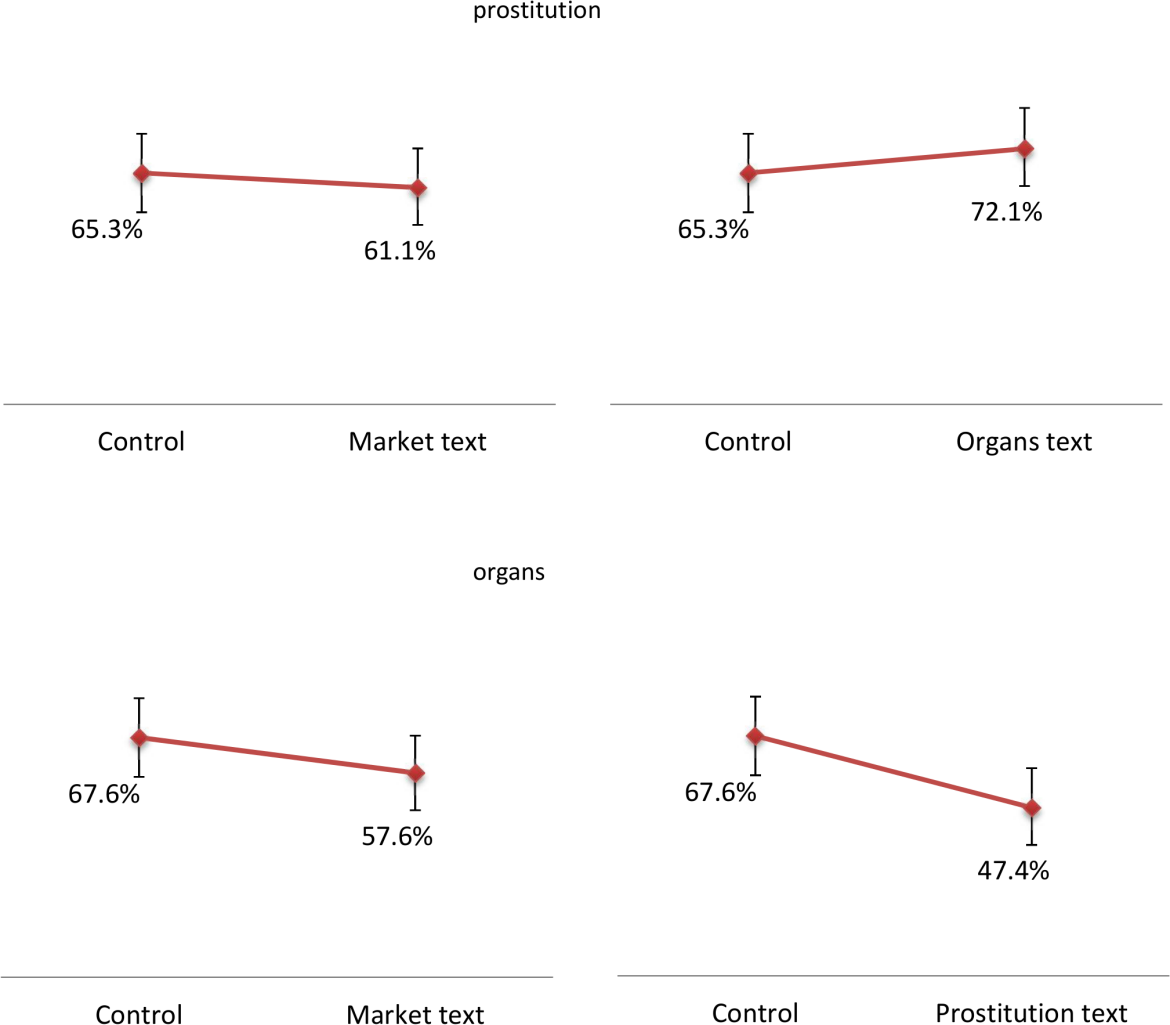
Estimated treatment effects. Notes: The figures report the percentage of subjects favoring either payments for organs or legalizing indoor prostitution, by treatment condition. The values were calculated as the differences between the average number of statements with which the subjects were in agreement when provided with five statements (including the statement about support for payment for organs/indoor prostitution) and the average number of statements with which the subjects were in agreement when provided with four statements (excluding the statement about support for payment for organs/indoor prostitution). The vertical bars indicate 95% confidence intervals for the estimates.

The estimated support rates for each condition are illustrated in Fig 2. The baseline support for legalizing organ payments and indoor prostitution (i.e. for subjects who did not read any text) was 67.6% and 65.3%, respectively. The approval rate for organ payments was higher than what found in previous studies [17,32]. This is most likely due to different sampling, and it is not the main focus of this study because we are interested in the *difference* between the baseline and the support after receiving additional information.

**Fig 3:**
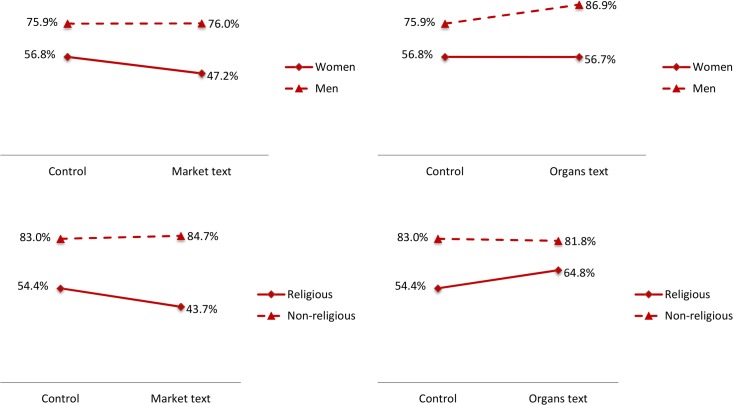
Estimated support for legalizing indoor prostitution, by gender and religiosity. Notes: The figures report the percentage of subjects favoring either payments for organs or legalizing indoor prostitution, by treatment condition and demographic trait. See notes to Fig 2 for details.

None of our treatments had a meaningful effect on the support for legalizing indoor prostitution. Reading a text with general considerations about the efficiency properties of market systems reduced support for legalizing prostitution by four percentage points (top-left panel of Fig 2; p>0.6). The text regarding the organ shortage and methods to solve it (including a price mechanism) increased support for indoor prostitution by 7 percentage points (top-right panel; p>0.4). In our companion study we also found no direct aggregate effect of a text about the potential reduction in violence and STDs associated with legalized indoor prostitution on support for its legalization [18]. These results suggest that US residents’ opinions regarding indoor prostitution are relatively stable, and are not affected by cost-benefit considerations. The relatively high baseline approval rate indicates that, presumably, individuals are already informed of the relevant facts, and that any residual opposition to legalizing prostitution is due to deeply-held moral beliefs. However, in line with our previous work and other studies, we find substantial heterogeneities along two features of the subjects: gender and religious attitudes. As shown in Fig 3, women and religious individuals reported substantially lower approval for legalizing prostitution compared to men, and these differences were consistent across experimental conditions. Moreover, note that the directionally negative effect of the market text on attitudes toward prostitution is entirely driven by women and religious individuals.

**Fig 4:**
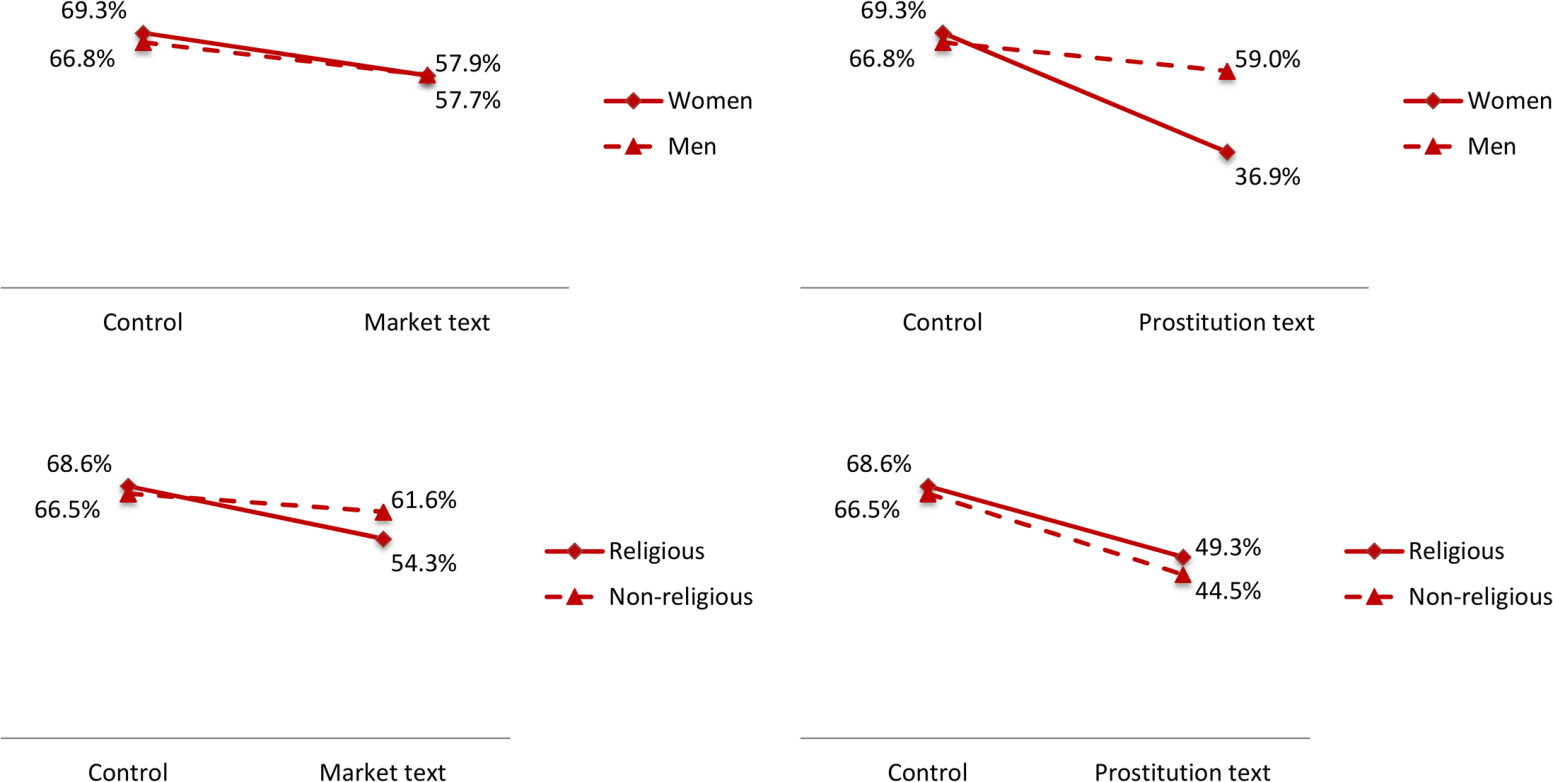
Estimated support for legalizing organ payments, by gender and religiosity. Notes: The figures report the percentage of subjects favoring either payments for organs or legalizing indoor prostitution, by treatment condition and demographic trait. See notes to Fig 2 for details.

As for support for organ payments, Fig 2 shows that the market text was associated with a statistically insignificant 10 percentage-point reduction in support for legalized payments to organ donors or their families (bottom-left panel; p>0.19), whereas reading a text about the potential reduction in violence and STDs associated with legalized indoor prostitution reduced support for organ payments by a large and statistically significant 20 percentage points (bottom-right panel; p<0.05). The heterogeneity analysis shown in Fig 4 reveals that the latter effect was largely due by women’s dramatic reduction in support for legalizing organ payments after reading the prostitution text (from 69% to 37%). This finding, again, is consistently with what we previously found with respect to the “direct” effect of information about legalizing prostitution on support for this activity for women [18], as well as with existing literature documenting the deep opposition of women toward a market for sex, plausibly triggered by associations of prostitution with stigmatization, sexual dominance and women’s oppression [33,34]. The bottom panel shows similar reactions of religious and non-religious individuals. The top-right and bottom-right panels of Fig 4 reveal a lack of heterogeneous effects of the generic text on the efficiency properties of market systems on attitudes toward legalizing organ payments, again mirroring the findings from our companion paper where very limited heterogeneity was found for the direct effects of information on the organ shortage and potential solutions on attitudes toward introducing a price mechanism in this context [18]. As shown in Tables 3, 4 and 5, none of our estimates meaningfully changed in regressions that included covariates for the socio-economic characteristics of the respondents and wave dummies to control for underlying trends.

**Table 3:** Regression estimates.

	Outcome variable: N. of statements that apply to subject			
	(1)	(2)	(3)	(4)
	Support for legalizing prostitution		Support for legalizing organ payments	
Covariates:	Market text	Organs text	Market text	Prostitution text
Constant	0.935	0.140	2.335***	2.268***
	(0.717)	(0.578)	(0.466)	(0.522)
Control (no text, 5 statements)	0.672***	0.678***	0.676***	0.672***
	(0.056)	(0.056)	(0.056)	(0.056)
Treatment, 4 statements	0.095	0.013	0.123	0.085
	(0.075)	(0.053)	(0.078)	(0.053)
Treatment, 5 statements	0.704***	0.714***	0.725***	0.563***
	(0.081)	(0.057)	(0.082)	(0.058)
Treatment: 5 statements—4 statements (share in favor of legalization in treatment condition)	0.609***	0.701***	0.603***	0.478***
	(0.056)	(0.059)	(0.054)	(0.059)
5 statements—4 statements: Treatment—control (difference in share in favor of legalization between treatment and control conditions)	-0.063	0.023	-0.074	-0.193**
	(0.079)	(0.082)	(0.078)	(0.082)
R-squared	0.176	0.196	0.163	0.151
Obs.	2,213	2,093	2,256	2,014

Notes: estimates are from ordinary least square regressions that include indicators for gender, job status, income level, educational attainment, relationship status, whether the respondent has children, monthly income, political views, religious beliefs, whether the subject donated to charity or volunteered in the previous two years, state of residence, as well as age in linear and quadratic value. An indicator for the second intervention wave is also added. Huber-White robust standard errors in parentheses.

**p<0.05;

***P<0.01.

**Table 4:** Regression estimates, heterogeneous effects: Support for legalizing prostitution.

	Market text			Organs text		
	Baseline support (no text)	Support after reading text	Effect of reading text	Baseline support (no text)	Support after reading text	Effect of reading text
Overall	67.2 (5.6)***	60.9 (5.6)***	*-6*.*3 (7*.*9)*	67.8 (5.6)***	70.1 (5.9)***	*2*.*3 (8*.*2)*
Women	58.6 (8.0)***	47.9 (7.4)***	*-10*.*6 (10*.*9)*	58.8 (8.0)***	54.8 (8.1)***	*-4*.*0 (11*.*4)*
Men	75.4 (8.0)***	74.5 (8.4)***	*-0*.*9 (11*.*5)*	76.5 (8.0)***	86.2 (8.5)***	*9*.*8 (11*.*6)*
*Difference Women—Men*	*-16*.*8 (11*.*3)*	-26.5 (11.1) **	*-9*.*7 (15*.*9)*	*-17*.*7 (11*.*3)*	*-31*.*5 (11*.*7* ***	*-13*.*8 (16*.*2)*
Religious	57.2 (7.7)***	45.3 (7.4)***	*-11*.*9 (10*.*6)*	*57*.*9 (7*.*7)* ***	63.0 (8.1)***	*5*.*1 (11*.*2)*
Non-religious	81.0 (8.3)***	83.3 (8.5)***	*2*.*3 (11*.*9)*	*81*.*2 (8*.*2)* ***	79.8 (8.3)***	*-1*.*4 (11*.*7)*
*Difference Relig*.*—Non relig*.	-23.8 (11.3) **	*-38*.*0 (11*.*3* ***	*14*.*3 (15*.*0)*	-23.3 (11.3)**	*-16*.*9 (11*.*6)*	*6*.*5 (16*.*1)*

Notes: estimates are from ordinary least square regressions that include indicators for gender, job status, income level, educational attainment, relationship status, whether the respondent has children, monthly income, political views, religious beliefs, whether the subject donated to charity or volunteered in the previous two years, state of residence, as well as age in linear and quadratic value. An indicator for the second intervention wave is also added. Huber-White robust standard errors in parentheses.

**p<0.05;

***P<0.01.

**Table 5:** Regression estimates, heterogeneous effects: Support for legalizing organ payments.

	Market text			Prostitution text		
	Baseline support (no text)	Support after reading text	Effect of reading text	Baseline support (no text)	Support after reading text	Effect of reading text
Overall	67.6 (5.6)***	60.3 (5.4) ***	*-7*.*4 (7*.*8)*	67.2 (5.6)	47.9 (5.9) ***	-19.3 (8.2)**
Women	69.4 (8.1) ***	61.9 (7.3) ***	*-7*.*4 (11*.*0)*	*67*.*9 (8*.*1)* ***	35.5 (8.0) ***	*-32*.*4 (11*.*3)* ***
Men	66.0 (7.9) ***	58.5 (9.9) ***	*-7*.*5 (11*.*2)*	*66*.*7 (7*.*9)* ***	61.0 (8.6) ***	*-5*.*7 (11*.*7)*
*Difference Women—Men*	*3*.*4 (10*.*8)*	*3*.*4 (10*.*8)*	*0*.*0 (15*.*7)*	*1*.*2 (11*.*4)*	25.4 (11.8) **	*26*.*7 (16*.*3)*
Religious	68.2 (7.6) ***	59.1 (7.0) ***	*-9*.*1 (10*.*4)*	*68*.*7 (7*.*7)* ***	47.1 (7.8) ***	-21.6 (10.9) **
Non-religious	67.3 (8.5) ***	61.9 (8.4) ***	*-5*.*3 (11*.*9)*	*65*.*3 (8*.*4)* ***	48.6 (9.0) ***	*-16*.*7 (12*.*3)*
*Difference Relig*.*—Non relig*.	*0*.*9 (11*.*5)*	*2*.*8 (10*.*9)*	*3*.*7 (15*.*8)*	*3*.*4 (11*.*4)*	*-1*.*5 (11*.*8)*	*-4*.*9 (16*.*7)*

Notes: estimates are from ordinary least square regressions that include indicators for gender, job status, income level, educational attainment, relationship status, whether the respondent has children, monthly income, political views, religious beliefs, whether the subject donated to charity or volunteered in the previous two years, state of residence, as well as age in linear and quadratic value. Wave indicators are also included. Huber-White robust standard errors in parentheses.

*p<0.1;

**p<0.05;

***p<0.01.

**Fig 5:**
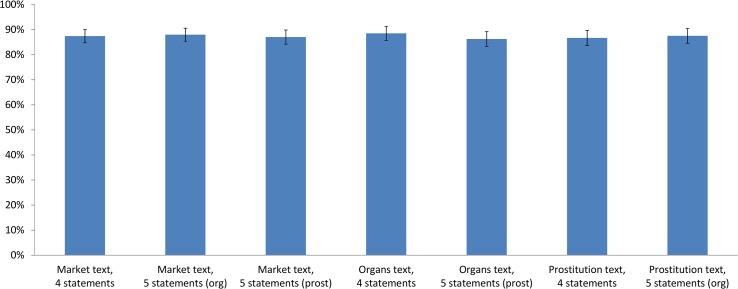
Percentage of subjects who found the provided text to be reliable, by treatment condition.

Finally, about 90% of the subjects found the text reliable in all treatment conditions (Fig 5). It is therefore unlikely that the perceived credibility of the information affected our results.

## Discussion

Two main insights can be derived from our analyses. First, general information (or additional focus) on the efficiency properties of markets did not affect individual attitudes toward the introduction of markets or price-mediated transactions in such morally controversial cases as organ donation and prostitution. This contrasts with the positive effect of providing context-specific information and a focus on the effects of markets in particular cases [18]. Moreover, the null effects of general market information (and if any, some directionally negative effects) are inconsistent with an increased focus on markets necessarily relaxing or corrupting the moral constraints in which people believe [9,19]. On the contrary, for some groups, such as women and religious individuals, it had a negative effect, making them less favorable to the use of markets. Second, we found some evidence of “spillover” effects of information on a morally controversial transaction to another, at least in a case where the two considered activities are somewhat related to the trade of the human body or body parts; however, these spillovers varied according to individual characteristics, such as gender, and other deep beliefs, such as religion.

These results imply that the provision of well-supported information can change attitudes toward the acceptance of morally charged market trades but it has be context-specific, thus potentially leading to implement, or at least experiment, as recently proposed [35,36], some market-based solutions to socially pressing issues such as the shortage organs for transplant. Because of the different responses for different activities, and interactions between attitudes, a case-by-case approach appears to be preferable for both scholars and policymakers in exploring responses to the introduction of these market-based solutions.

## Supporting Information

S1 Table Descriptive statistics on individual characteristics collected from the survey, overall and by experimental condition. Notes: (*) 1 = No text, 4 statements; 2 = No text, 5 statements (organ payments); 3 = No text, 5 statements (prostitution); 4 = Organs text, 4 statements; 5 = Organs text, 5 statements (prostitution); 6 = Prostitution text, 4 statements; 7 = Prostitution text, 5 statements (organ payments); 8 = Market text, 4 statements; 9 = Market text, 5 statements (organ payments); 10 = Market text, 5 statements (prostitution).
(DOCX)

S2 Table Comparison between the mTurk sample and the US population on selected socio-economic characteristics. Notes: Figures on the US population are from Leider and Roth (2011). (*) Due to missing values, the number of observations is 5,054 for political orientation.
(DOCX)

S1 Text Experimental material. (DOCX)
