# Supplementary material for: Markets and Morals: An Experimental Survey Study
Source: PLoS One. 2015 Jun 1;10(6):e0127069. doi: 10.1371/journal.pone.0127069 (PMC4451523; doi:10.1371/journal.pone.0127069)
Supplement: S1 Table — Notes: (*) 1 = No text, 4 statements; 2 = No text, 5 statements (organ payments); 3 = No text, 5 statements (prostitution); 4 = Organs text, 4 statements; 5 = Organs text, 5 statements (prostitution); 6 = Prostitution text, 4 statements; 7 = Prostitution text, 5 statements (organ payments); 8 = Market text, 4 statements; 9 = Market text, 5 statements (organ payments); 10 = Market text, 5 statements (prostitution). (DOCX) [file pone.0127069.s001.docx]

Table S1: Descriptive statistics on individual characteristics collected from the survey, overall and by experimental condition.

|  |  |  |  |  |  |  |  |  |  |  |
| --- | --- | --- | --- | --- | --- | --- | --- | --- | --- | --- |
| Experimental conditions^(*)^: | **1** | **2** | **3** | **4** | **5** | **6** | **7** | **8** | **9** | **10** |
|  | Average age | | | | | | | | | |
|  | 32.0 | 32.5 | 32.5 | 32.7 | 33.3 | 34.1 | 32.6 | 32.0 | 31.9 | 32.0 |
|  | Gender | | | | | | | | | |
| Women | 53.2% | 49.4% | 47.7% | 47.2% | 50.7% | 47.9% | 47.2% | 49.5% | 48.1% | 48.7% |
| Men | 46.8% | 50.6% | 52.3% | 52.8% | 49.3% | 52.1% | 52.8% | 50.5% | 51.9% | 51.3% |
|  | Ethnicity | | | | | | | | | |
| White/Caucasian | 74.7% | 77.0% | 77.7% | 77.2% | 80.3% | 77.7% | 79.0% | 77.5% | 77.9% | 79.1% |
| African American | 9.1% | 6.5% | 8.0% | 7.7% | 6.0% | 7.1% | 8.3% | 9.2% | 9.1% | 6.9% |
| Hispanic | 5.5% | 7.0% | 6.0% | 5.0% | 5.0% | 4.8% | 5.2% | 3.9% | 5.3% | 3.9% |
| Asian | 8.1% | 7.6% | 5.4% | 6.9% | 5.8% | 7.3% | 5.0% | 6.7% | 5.4% | 7.4% |
| Other | 2.6% | 1.9% | 2.9% | 3.2% | 2.9% | 3.1% | 2.5% | 2.7% | 2.3% | 2.8% |
|  | Educational attainment | | | | | | | | | |
| Primary School | 0.6% | 0.2% | 0.2% | 0.4% | 0.8% | 0.2% | 0.4% | 0.2% | 0.0% | 0.0% |
| Some high school | 4.5% | 4.6% | 4.5% | 3.6% | 4.6% | 2.5% | 1.9% | 1.0% | 0.8% | 0.9% |
| High School | 19.6% | 18.4% | 17.8% | 18.5% | 20.3% | 16.7% | 18.9% | 8.7% | 8.2% | 9.8% |
| Some university | 34.9% | 39.2% | 37.4% | 37.3% | 38.3% | 37.1% | 40.1% | 38.3% | 43.7% | 38.5% |
| Undergraduate univ. degree | 32.3% | 29.1% | 30.5% | 29.4% | 28.0% | 33.1% | 27.0% | 34.7% | 34.6% | 36.1% |
| Postgraduate degree | 8.1% | 8.6% | 9.6% | 10.7% | 7.9% | 10.4% | 11.6% | 17.1% | 12.7% | 14.6% |
|  | Job status | | | | | | | | | |
| Private employee | 34.9% | 34.0% | 33.2% | 35.9% | 35.8% | 40.4% | 37.0% | 33.2% | 31.1% | 31.3% |
| Public employee | 21.7% | 19.0% | 16.5% | 15.3% | 14.1% | 17.5% | 16.0% | 15.9% | 16.0% | 17.0% |
| Self employed/ entrepreneur | 14.2% | 15.0% | 16.5% | 15.7% | 18.8% | 14.0% | 17.7% | 17.3% | 16.0% | 16.7% |
| Unemployed | 11.7% | 12.0% | 10.3% | 12.1% | 12.0% | 12.1% | 8.5% | 9.9% | 12.2% | 10.4% |
| Housekeeper | 5.8% | 4.9% | 6.9% | 6.9% | 7.0% | 4.2% | 6.4% | 4.4% | 4.4% | 4.8% |
| Student | 10.0% | 12.2% | 14.7% | 12.1% | 9.5% | 10.0% | 11.6% | 17.3% | 18.5% | 17.6% |
| Retired | 1.7% | 2.9% | 1.8% | 2.0% | 2.9% | 1.9% | 2.7% | 2.0% | 1.8% | 2.2% |
|  | Monthly income | | | | | | | | | |
| No income | 13.8% | 12.4% | 13.4% | 12.9% | 12.0% | 12.1% | 12.5% | 14.9% | 16.8% | 13.9% |
| Less than $1,500 | 32.8% | 35.6% | 35.0% | 32.7% | 37.5% | 30.2% | 32.0% | 29.5% | 34.9% | 35.0% |
| Between $1,500 and $2,500 | 24.5% | 25.3% | 22.3% | 24.8% | 23.6% | 28.8% | 25.2% | 25.5% | 19.9% | 21.5% |
| Between $2,500 and $5,000 | 20.0% | 18.8% | 23.0% | 23.6% | 20.5% | 22.9% | 23.7% | 20.3% | 21.3% | 19.6% |
| More than $5,001 | 8.9% | 8.0% | 6.2% | 6.0% | 6.4% | 6.0% | 6.7% | 9.7% | 7.1% | 10.0% |

Continues on the next page

Table SI1 – continued from previous page

|  |  |  |  |  |  |  |  |  |  |  |
| --- | --- | --- | --- | --- | --- | --- | --- | --- | --- | --- |
| Experimental conditions^(*)^: | **1** | **2** | **3** | **4** | **5** | **6** | **7** | **8** | **9** | **10** |
|  | Relationship status | | | | | | | | | |
| Single | 37.4% | 33.1% | 38.3% | 38.1% | 34.6% | 35.2% | 35.6% | 35.7% | 38.2% | 34.4% |
| Unmarried in a relationship | 25.8% | 23.8% | 18.9% | 23.6% | 25.1% | 21.0% | 22.9% | 23.2% | 21.4% | 27.2% |
| Married | 31.1% | 35.4% | 36.8% | 31.9% | 33.7% | 35.6% | 35.6% | 36.2% | 34.1% | 33.0% |
| Separated/divorced | 5.3% | 6.7% | 6.0% | 5.4% | 5.6% | 7.5% | 5.6% | 4.2% | 5.6% | 4.8% |
| Other | 0.4% | 1.1% | 0.0% | 1.0% | 1.0% | 0.6% | 0.4% | 0.7% | 0.7% | 0.6% |
|  | Children | | | | | | | | | |
| Has children | 36.4% | 40.7% | 37.9% | 39.1% | 38.9% | 39.8% | 41.2% | 35.6% | 32.3% | 35.9% |
| Does not have children | 63.6% | 59.3% | 62.1% | 60.9% | 61.1% | 60.2% | 58.8% | 64.4% | 67.7% | 64.1% |
|  | Political views | | | | | | | | | |
| Conservative | 16.0% | 18.8% | 20.5% | 16.3% | 16.4% | 19.6% | 18.3% | 20.8% | 18.8% | 18.3% |
| Liberal | 44.7% | 44.3% | 39.2% | 46.8% | 47.4% | 43.8% | 44.5% | 40.1% | 42.7% | 47.0% |
| Moderate | 33.4% | 31.9% | 35.8% | 31.9% | 32.1% | 31.9% | 32.2% | 33.2% | 33.4% | 29.3% |
| Other | 5.8% | 4.9% | 4.5% | 5.0% | 4.1% | 4.8% | 5.0% | 5.9% | 5.1% | 5.4% |
|  | Religious beliefs | | | | | | | | | |
| Atheist/agnostic | 44.2% | 37.5% | 38.5% | 42.3% | 42.6% | 40.9% | 37.1% | 40.7% | 41.4% | 41.6% |
| Christian | 45.1% | 50.3% | 47.5% | 45.6% | 42.6% | 46.3% | 51.9% | 46.0% | 45.6% | 45.5% |
| Jewish | 1.3% | 1.5% | 2.2% | 2.2% | 1.7% | 1.9% | 1.3% | 1.5% | 2.0% | 1.7% |
| Muslim | 8.7% | 10.1% | 10.9% | 8.9% | 12.6% | 9.4% | 9.6% | 10.9% | 10.2% | 10.2% |
| Other | 0.8% | 0.6% | 0.9% | 1.0% | 0.6% | 1.5% | 0.2% | 0.8% | 0.8% | 1.1% |
|  | Charitable/volunteering behavior | | | | | | | | | |
| Donated /volunteered in past 2 years | 35.7% | 32.7% | 35.0% | 31.0% | 32.3% | 29.4% | 29.1% | 25.3% | 27.2% | 25.0% |
| Did not Donate /volunteer in past 2 years | 64.3% | 67.3% | 65.0% | 69.0% | 67.7% | 70.6% | 70.9% | 74.7% | 72.8% | 75.0% |

Notes: (*) 1 = No text, 4 statements; 2 = No text, 5 statements (organ payments); 3 = No text, 5 statements (prostitution); 4 = Organs text, 4 statements; 5 = Organs text, 5 statements (prostitution); 6 = Prostitution text, 4 statements; 7 = Prostitution text, 5 statements (organ payments); 8 = Market text, 4 statements; 9 = Market text, 5 statements (organ payments); 10 = Market text, 5 statements (prostitution).

Table SI2: Comparison between the mTurk sample and the US population on selected socio-economic characteristics.

|  |  |  |  |
| --- | --- | --- | --- |
|  | mTurk  sample |  | US population |
| Age | 32.5 |  | 44.3 |
| % Female | 49.0% |  | 52.4% |
| % Caucasian | 77.8% |  | 68.2% |
| % Black | 7.8% |  | 11.6% |
| % Other ethnicity | 14.4% |  | 20.2% |
| % Married | 34.4% |  | 54.1% |
| % College degree | 49.8% |  | 33.0% |
| % Christian faith | 46.5% |  | 76% |
| % Conservative | 19.4% |  | 39% |
| % Liberal | 46.3% |  | 32% |
| N | 5,324 |  |  |
|  |  |  |  |

Notes: Figures on the US population are from Leider and Roth (2011). (*) Due to missing values, the number of observations is 5,054 for political orientation.
