# Supplementary material for: Markets and Morals: An Experimental Survey Study
Source: PLoS One. 2015 Jun 1;10(6):e0127069. doi: 10.1371/journal.pone.0127069 (PMC4451523; doi:10.1371/journal.pone.0127069)
Supplement: S2 Table — Notes: Figures on the US population are from Leider and Roth (2011). (*) Due to missing values, the number of observations is 5,054 for political orientation. (DOCX) [file pone.0127069.s002.docx]

Table S2: Comparison between the mTurk sample and the US population on selected socio-economic characteristics.

|  |  |  |  |
| --- | --- | --- | --- |
|  | mTurk  sample |  | US population |
| Age | 32.5 |  | 44.3 |
| % Female | 49.0% |  | 52.4% |
| % Caucasian | 77.8% |  | 68.2% |
| % Black | 7.8% |  | 11.6% |
| % Other ethnicity | 14.4% |  | 20.2% |
| % Married | 34.4% |  | 54.1% |
| % College degree | 49.8% |  | 33.0% |
| % Christian faith | 46.5% |  | 76% |
| % Conservative | 19.4% |  | 39% |
| % Liberal | 46.3% |  | 32% |
| N | 5,324 |  |  |
|  |  |  |  |

Notes: Figures on the US population are from Leider and Roth (2011). (*) Due to missing values, the number of observations is 5,054 for political orientation.
