# Supplementary material for: Markets and Morals: An Experimental Survey Study
Source: PLoS One. 2015 Jun 1;10(6):e0127069. doi: 10.1371/journal.pone.0127069 (PMC4451523; doi:10.1371/journal.pone.0127069)
Supplement: S1 Text — (DOCX) [file pone.0127069.s003.docx]

**S1 (Text): Experimental material**

**S1A - Treatment: Market Text**

**PART 1: TEXT**

**The role of markets in modern society**

Economists and policymakers often disagree as of what the preferred solution to an economic or social problem is. However, the widespread agreement, at least in the Western world since the work of Adam Smith in the eighteenth century, is that a market system based on a price mechanism is a good way to organize most parts of the economy and can achieve higher welfare than other historically experimented systems (1).

There are different opinions as to the extent to which the government should intervene in markets, for example to limit monopolies (2) or control pollution (3) or to assure a minimum provision of goods considered necessary for a decent life (4). However, in most circumstances of ordinary life, there are some characteristics of the functioning of the price mechanism that make it an attractive way of organizing exchanges of goods and services. Some of the properties of markets, and of the price system that derives from the free interaction of buyers and suppliers, can be summarized as follows.

First, markets provide producers with incentives to provide goods and services that consumers want, and individuals with incentives to acquire useful skills, i.e. skills that are requested by firms, organizations, the government, and so on.

Second, markets transmit information, through prices, about the scarcity of goods and resources. Prices serve as signal of the desirability, need, and scarcity of a given good, service or resource, helping to bring demand into balance with supply. When goods are allocated using monetary prices, the cost paid by buyers equals the revenue obtained by sellers; therefore no resources are lost in the process of equating supply and demand. With other market-clearing mechanisms, such as queues, consumers incur a cost in terms of waiting time, with no corresponding revenue to sellers (5).

Third, market competition operates as a disciplinary device pushing businesses to keep costs down. To the extent that markets are institutionalized and at least partially regulated (e.g. by requiring safety or health standards, or certain qualifications to operate as suppliers), markets and prices combined with appropriate taxes may also lead to a more efficient organization and control of trades that would occur illegally in the underground economy otherwise (6).

Finally, the market system involves a high degree of economic freedom (7): The freedoms of choice, property and enterprise are more likely to be fulfilled in a system where the price mechanism operates.

(1)     See, for example, Chapter 2: “Markets and Government in a Modern Economy” of Samuelson, Paul and William Nordhaus, 2004. “Economics,” McGraw-Hill/Irwin; 18th edition.

(2)     Tirole, J., 1988. The Theory of Industrial Organization. Cambridge: MIT Press.

(3)     Laffont, Jean J., 2008. “Externalities,” The New Palgrave Dictionary of Economics, Second Edition, 2008

(4)     Sen, Amartya. 1989. “The Standard of Living,” Cambridge University Press.

(5)     See, for example, Nicholson, Walter and Christopher Snyder, 2011. “Microeconomic Theory: Basic Principles and Extensions,” Cengage Learning; 11 edition.

(6)     Becker, Gary S., Kevin M. Murphy and Michael Grossman, “ Market for Illegal Goods: The Case of Drugs,” Journal of Political Economy, 114(1), págs. 38-60, 2006.

(7)     Friedman, Milton, 1962. “Capitalism and Freedom,” Chicago: University of Chicago Press.

**PART 2: Comprehension Question**

Based on the article that you just read, please answer the following question:

Please briefly summarize one of the properties of market economies, among those described in the text.

**PART 3: Elicitation of attitudes**

Four statements condition:

Please report below how many of the following statements apply to you

1. For my financial decisions, I usually use the services of a financial adviser.
2. I voted or would consider voting for a congressional candidate who supports “pro-choice” policies.
3. I normally file my tax return on my own, using pre-packaged software or paper forms.
4. I would not support federal legislation aimed at restricting the possession of firearms.

Five statement condition/Organ payments

Please report below how many of the following statements apply to you

1. For my financial decisions, I usually use the services of a financial advisor.
2. I normally file my tax return on my own, using pre-packaged software or paper forms.
3. I voted or would consider voting for a congressional candidate who supports “pro-choice” policies.
4. I would not support federal legislation aimed at restricting the possession of firearms.
5. I would support the introduction of regulated monetary payments for live organ donors and for the families of deceased organ donors.

(Note: the order of the statements was randomized)

Five statement condition/Indoor prostitution

Please report below how many of the following statements apply to you

1. For my financial decisions, I usually use the services of a financial advisor.
2. I normally file my tax return on my own, using pre-packaged software or paper forms.
3. I voted or would consider voting for a congressional candidate who supports “pro-choice” policies.
4. I would not support federal legislation aimed at restricting the possession of firearms.
5. I would support the legalization of indoor prostitution within a regulated framework.

(Note: the order of the statements was randomized)

**PART 4: Reliability**

Please answer the following question.

Did you consider the information provided in the text you read at the beginning of this survey reliable?

(Yes/No)

**S1B - Treatment: Organs Text**

**PART 1: TEXT**

**The Current Situation of the Kidney Transplant System**

In 2013, 99,500 Americans were on the waiting list for a new kidney (1). Yet, only about 16,900 kidney transplant operations were performed in that year. The current average waiting time for a kidney transplant is 4.7 years, up from about 2.9 years only a decade ago (2). A significant increase in the supply of kidneys available for transplantation is required to reduce waiting times.

Kidneys for transplantation come from either deceased or living donors (people who can remain healthy with only one kidney). Finding a compatible kidney is not easy. There are four basic blood types, and tissue matching involves the combination of six proteins. Blood and tissue type determine the likelihood that a kidney will help a recipient in the long run.

Most organ procurements efforts in the U.S. have focused on increasing the pool of altruistic donors, e.g. by expanding living donor inclusion criteria and through organ donor awareness campaigns. In recent years, kidney exchanges—in which pairs of living would-be donors and recipients who prove incompatible look for another pair or pairs of donors and recipients who would be compatible for transplants, cutting their wait time—have become more widespread. In 2013, 590 kidney transplants were performed through these exchanges, accounting for 10% of live donations and 3.5% of all kidney transplants, including those with after-death donations.

In an attempt to increase cadaveric organ donations, some nations have enacted the principle of “implied consent”, whereby organs from cadavers are assumed to be available for transplant unless, before death, individuals indicate that they don't want their organs to be used. However, even with the capture of all potential deceased donor organs, the gap will not be closed. In 2013, the demand for kidney transplants was 25,500 (3). To satisfy demand, the system would need to generate about 50 deceased donors per million people (pmp). Today, the donation rate is 26 pmp (4).

Another proposal to increase the supply of kidneys is to provide compensation to living donors and/or to the families of individuals who agree in advance to donate a kidney after they die, within a regulated framework. According to some studies (5), a sufficient payment to kidney donors could increase the supply of kidneys by a large percentage. Estimates that consider the risk to donors from transplant surgery, the number of weeks of work lost during the surgery and recovery periods, and the risk of reduction in the quality of life, indicate that with a payment of about US$15,000 for a kidney (6), the total number of kidney transplants would increase from 16,900 to 23,10 (7).

(1) Unless indicated otherwise, all numbers on the kidney transplant system are from the Organ Procurement and Transplantation Network (OPTN) of the United States.

(2) Using data from OPTN, the average waiting time is computed by dividing the number of patients in the waiting list by the total number of transplants and the number of deaths on the waiting list.

(3) Using data from OPTN, the annual demand is computed as the total number of kidney transplants plus the growth in the waiting list and number of death in the waiting list.

(4) RODaT: The International Online Registry for Organ Donation and Transplantation.

(5) T. Randolph Beard, David L. Kaserman, and RigmarOsterkamp. “The Global Organ Shortage Economic Causes, Human Consequences, Policy Responses,” Stanford University Press, 2013.

(6) Becker, Gary S. and Julio J. Elías. “Introducing Incentives in the Market for Live and Cadaveric Organ Donations,” Journal of Economic Perspectives, Summer 2007.

(7) Updated calculations following Becker and Elías, 2007.

**PART 2: Comprehension question**

Based on the article that you just read, please answer the following question

Please report the price that some studies have identified to potentially compensate kidney donors, and how much they estimate this would increase the supply of kidneys.

**PART 3: Elicitation of attitudes**

Four statements condition:

Please report below how many of the following statements apply to you

1. For my financial decisions, I usually use the services of a financial advisor.
2. I normally file my tax return on my own, using pre-packaged software or paper forms.
3. I voted or would consider voting for a congressional candidate who supports “pro-choice” policies.
4. I would not support federal legislation aimed at restricting the possession of firearms.

Five statement condition/Indoor prostitution

Please report below how many of the following statements apply to you

1. For my financial decisions, I usually use the services of a financial advisor.
2. I normally file my tax return on my own, using pre-packaged software or paper forms.
3. I voted or would consider voting for a congressional candidate who supports “pro-choice” policies.
4. I would not support federal legislation aimed at restricting the possession of firearms.
5. I would support the legalization of indoor prostitution within a regulated framework.

(Note: the order of the statements was randomized)

**PART 4: Reliability**

Please answer the following question.

Did you consider the information provided in the text you read at the beginning of this survey reliable?

(Yes/No)

**S1C - Treatment: Prostitution Text**

**PART 1: TEXT**

**Prostitution in the United States**

Prostitution in the United States is illegal, except in some rural counties in Nevada. The prohibition is enforced in different forms, such as incarcerating sex workers, charging individuals engaging in prostitution with a felony, or implementing prostitution diversion programs. Prostitution, however, is present in most parts of the country, in various forms. In the last 15 years, the American prostitution market has shifted from a primarily outdoor (street-based) to an indoor market (massage parlors, escort agencies, and much of the online activity) (1). The prostitution trade is estimated to gross over $14 billion a year in the US (2). A 2004 poll reports that 30% of single men over the age of 30 have paid for sex in the US (3).

Disease transmission and victimization risks are associated with sex markets. The 1992 National Health and Social Life Survey (NHSLS) reports that 22.9% of female prostitutes report they have had gonorrhea relative to 4.7% of non-prostitute females. The spread of this and other sexually transmitted diseases is a public health concern (4). Sex market-related violence is also common. One study finds that 68% of women engaged in street-level prostitution have been raped by clients and another reports that one third of all serial murder victims are prostitutes (5).

To reduce the potential costs associated with prostitution, some analysts have proposed decriminalizing indoor sex work, but few governments have been willing to experiment with the policy. It has been argued that indoor prostitution typically involves less exploitation, less risk of violence, more control over working conditions, more job satisfaction, and higher self-esteem (6). Street prostitution has higher rates of gonorrhea (7), rape and sexual assault than indoor prostitution (8).

A recent study (9) finds that the decriminalization of indoor prostitution in Rhode Island in 2003 caused both forcible rape offenses and gonorrhea incidence to decline for the overall population. From 2004 to 2009, reported rape offenses decreased by 31% and the number of cases of female gonorrhea decreased by 39%.

(1) Cunningham, Scott and Todd D. Kendall. 2001. “Prostitution 2.0: The Changing Face of Sex Work,” Journal of Urban Economics.

(2) Havoscope. 2013. http://www.havocscope.com/prostitution-revenue-by-country/.Technical report.

(3) Langer, Gary, Cheryl Arnedt and Dalia Sussman. 2004. \Primetime Live Poll: American Sex Survey.”.

(4) National Institutes of Health, 2001.

(5) Farley, Melissa and Vanessa Kelly. 2000. “Prostitution: a critical review of the medical and social sciences literature,” Women and Criminal Justice.

(6) Weitzer, Ronald. 2005. “New Directions in Research on Prostitution,” Crime, Law & Social Change.

(7) Potterat, John J., Richard B. Rothenberg and Donald C. Bross. 1979. “Gonorrhea in street prostitutes: epidemiologic and legal implications,” Sexually Transmitted Diseases.

(8) Church, Stephanie, Marion Henderson, Marina Barnard and Graham Hart. 2001. “Violence by clients towards female prostitutes in different work settings: questionnaire survey,” British Medical Journal.

(9) Cunningham, Scott and Shah, Manisha. 2014. “Decriminalizing Indoor Prostitution: Implications for Sexual Violence and Public Health,” NBER Working Papers, National Bureau of Economic Research.

**PART 2: Question**

Based on the article that you just read, please answer the following question

Please report the declines in rape offenses and cases of female gonorrhea estimated in the study described in the reading.

**PART 3: Elicitation of attitudes**

Four statements condition:

Please report below how many of the following statements apply to you

1. For my financial decisions, I usually use the services of a financial advisor.
2. I normally file my tax return on my own, using pre-packaged software or paper forms.
3. I voted or would consider voting for a congressional candidate who supports “pro-choice” policies.
4. I would not support federal legislation aimed at restricting the possession of firearms.

Five statement condition/Organ payments

Please report below how many of the following statements apply to you

1. For my financial decisions, I usually use the services of a financial advisor.
2. I normally file my tax return on my own, using pre-packaged software or paper forms.
3. I voted or would consider voting for a congressional candidate who supports “pro-choice” policies.
4. I would not support federal legislation aimed at restricting the possession of firearms.
5. I would support the introduction of regulated monetary payments for live organ donors and for the families of deceased organ donors.

(Note: the order of the statements was randomized)

**PART 4: Reliability**

Please answer the following question.

Did you consider the information provided in the text you read at the beginning of this survey reliable?

(Yes/No)

**1D - Questions common to all treatment conditions**

**What is your age in years?**

**Are you**

Male

Female

**What is your state of residence?**

**What is your race?**

White/Caucasian

African American

Hispanic

Asian

Other (specify)

**What is your relationship status?**

Single

Unmarried but in a relationship

Married

Separated/Divorced

Other

**What is your parental status?**

I have children

I do not have children

**What is your highest degree of education attained?**

Completed primary school

Some high school

Completed high school

Some university

Completed undergraduate university degree

Postgraduate

**What is your current employment status?**

Private Employee

Public Employee

Self-employed/Entrepreneur

Unemployed

Housekeeper

Student

Retired

**Approximately what is your monthly income?**

I am not currently earning any income

Less than $1.500

Between $1.500 and $2.500

Between $2.500 and $5.000

More than $5.000

**Have you donated money or volunteered time to a charitable organization in the past 2 years?**

No

Yes

**What is your religion?**

Atheist/Agnostic

Christian

Jewish

Muslim

Other

**What are your political views?**

Conservative

Liberal

Moderate

Other

**What you think the main objective of this survey was?**
